# Direct enzymatic ethanolysis of potential *Nannochloropsis* biomass for co-production of sustainable biodiesel and nutraceutical eicosapentaenoic acid

**DOI:** 10.1186/s13068-019-1418-7

**Published:** 2019-04-05

**Authors:** Yongjin He, Xiaofei Wang, Hehong Wei, Jianzhi Zhang, Bilian Chen, Feng Chen

**Affiliations:** 1 BIC-ESAT, College of Engineering, Peking University, Beijing, 100871 China; 2 Institute for Food & Bioresource Engineering, College of Engineering, Peking University, Beijing, 100871 China; 3 Key Laboratory of Feed Biotechnology, The Ministry of Agriculture of the People’s Republic of China, Beijing, 100081 China; 4 College of Life Science, Fujian Normal University, Fuzhou, 350117 China; 5 Institute for Advanced Study, Shenzhen University, Shenzhen, 518000 China

**Keywords:** *Nannochloropsis* sp. biomass, Ethanolysis, Biodiesel, Eicosapentaenoic acid, Enrichment

## Abstract

**Background:**

Marine microalga *Nannochloropsis* is a promising source for the production of renewable and sustainable biodiesel in replacement of depleting petroleum. Other than biodiesel, *Nannochloropsis* is a green and potential resource for the commercial production of nutraceutical eicosapentaenoic acid (EPA, C_20:5_). In recent studies, low-value biodiesel can be achieved by transesterification of *Nannochloropsis* biomass. However, it is undoubtedly wasteful to produce microalgal biodiesel containing EPA from nutritional and economical aspects. A new strategy was addressed and exploited to produce low-value bulky biodiesel along with EPA enrichment via enzymatic ethanolysis of *Nannochloropsis* biomass with a specific lipase.

**Results:**

Cellulase pretreatment on *Nannochloropsis* sp. biomass significantly improved the biodiesel conversion by direct ethanolysis with five enzymes from *Candida antarctica* (CALA and CALB), *Thermomyces lanuginosus* (TL), *Rhizomucor miehei* (RM), and *Aspergillus oryzae* (PLA). Among these five biocatalysts, CALA was the best suitable enzyme to yield high biodiesel conversion and effectively enrich EPA. After optimization, the maximum biodiesel conversion (46.53–48.57%) was attained by CALA at 8:1 ethanol/biomass ratio (v/w) in 10–15% water content with 10% lipase weight at 35 °C for 72 h. Meanwhile, EPA (60.81%) was highly enriched in microalgae NPLs (neutral lipids and polar lipids), increasing original EPA levels by 1.51-fold. Moreover, this process was re-evaluated with two *Nannochloropsis* species (IMET1 and Salina 537). Under the optimized conditions, the biodiesel conversions of IMET1 and Salina 537 by CALA were 63.41% and 54.33%, respectively. EPA contents of microalgal NPLs were 50.06% for IMET1 and 53.73% for Salina 537.

**Conclusion:**

CALA was the potential biocatalyst to discriminate against EPA in the ethanolysis of *Nannochloropsis* biomass. The biodiesel conversion and EPA enrich efficiency of CALA were greatly dependent on lipidic class and fatty acid compositions of *Nannochloropsis* biomass. CALA-catalyzed ethanolysis with *Nannochloropsis* biomass was a promising approach for co-production of low-value biodiesel and high-value microalgae products rich in EPA.

## Background

Microalgae feedstock is a promising source for the production of renewable and sustainable biodiesel in replacement of depleting petroleum [1–3]. Since marine microalgae *Nannochloropsis* can be cultivated with brackish or salty water in open raceway ponds without competition of arable land or freshwater supply [1, 4], it has attracted researchers’ attention for cost-effective biodiesel production via enzymatic method [1, 2, 5, 6]. López et al. [5] developed a direct enzymatic transesterification of *Nannochloropsis gaditana* B-3 biomass with immobilized lipase Novozym 435 (lipase B from *Candida antarctica*) for biodiesel. Law et al. [6] selected a soluble *Rhizomucor miehei* lipase for a single-step enzymatic process for directly transesterifying *Nannochloropsis salina* biomass to produce biodiesel. Enzymatic method is proved to be a mild, low-energy, environmentally friendly approach for microalgae-derived biodiesel production [1, 2].

Other than biodiesel, *Nannochloropsis* is a promising resource for the commercial production of nutraceutical eicosapentaenoic acid (EPA, C_20:5_) [1, 7–9]. EPA belongs to omega-3 polyunsaturated fatty acids (ω-3 PUFAs), which exhibits human beneficial functions such as anti-cancer and cardioprotective properties, which has been used in food and pharmaceutical applications [10–12]. Since fish oil has been detected with many harmful compounds [e.g., mercury (Hg) and pesticides] influenced by severe marine pollution in recent years, concerns about the safety of fish oil for EPA production have been voiced [13, 14]. It has been reported that *Nannochloropsis* species with individual fatty acid synthesis can produce green and sustainable EPA [1, 7, 8, 15]. However, as aforementioned, EPA of *Nannochloropsis* biomass is directly transesterified into biodiesel by enzymatic method [5, 6], which is undoubtedly wasteful and luxury from nutritional and economical aspects. Notably, EPA of *Nannochloropsis* lipids is effectively enriched by enzymatic transesterification with a specific lipase, resulting in the decrease in biodiesel conversion. To achieve the maximum biodiesel conversion and yield the highest EPA enrichment, it is important to develop a simple and efficient process to directly transesterify *Nannochloropsis* biomass.

Biodiesel production and PUFA enrichment have been achieved by versatile enzymes [16–20]. Microalgal biodiesel can be produced via lipase-catalyzed transesterification of extracted lipids or microalgae biomass using short-chain alcohols (e.g., methanol, ethanol, and 1-propanol, etc.) [1, 2, 6, 10, 16]. Meanwhile, PUFAs are effectively enriched by lipase-catalyzed hydrolysis or ethanolysis of fish or microalgae oil [16–21]. Compared with the hydrolysis reaction, ethanolysis for PUFAs’ enrichment have the following advantages [21–23]: (1) Ethanol acting as both substrate and reaction medium is the greenest solvent after water and has been widely applied in food industry. (2) High substrate conversion can be achieved. (3) Side product FAEEs is a clean-burning biodiesel. Thus, to enrich the PUFAs for food application, enzymatic ethanolysis is chosen in this study. To the best of our knowledge, this is the first study to simultaneously produce low-value bulky biodiesel and enrich EPA via enzymatic ethanolysis of *Nannochloropsis* biomass with a potential lipase.

In general, most biocatalysts from *C. antarctica*, *Thermomyces lanuginosus*, *R. miehei* (RM), and *Aspergillus oryzae* (PLA), etc. [16–20] are in liquid or immobilized form [10, 17, 18]. Expensive supporting materials and a series of high-energy and environmental-unfriendly procedures are usually used to immobilize enzymes, resulting in their high price [24]. On the contrary, the use of the liquid enzyme can lower the cost of an enzymatic process for oleochemicals with cheap price [10, 21, 24]. Price et al. [25] evaluated the economic viability of industrial biodiesel in a 40 m^3^ reactor using a liquid formulated enzyme and found that the cost of enzyme (one-time use) only accounted for less than 5% of the biodiesel revenue. Thus, enzymatic process mediated with liquid enzyme can be a promising and cost-effective approach for PUFA enrichment along with biodiesel production.

The aim of this study, thus, is to produce microalgal biodiesel and enrich EPA simultaneously via an efficient enzymatic process by a suitable liquid enzyme using *Nannochloropsis* biomass as raw material. First, five liquid enzymes from *C. antarctica* (CALA and CALB), *T. lanuginosus* (TL), *R. miehei* (RM), and *A. oryzae* (PLA) were selected to screen the best biocatalyst for EPA enrichment along with biodiesel production. Then, the reaction parameters including pretreatment of *Nannochloropsis* biomass, ethanol/biomass ratio (v/w), temperature, water content, enzyme weight, and reaction time in the enzymatic ethanolysis of *Nannochloropsis* biomass were deeply investigated to yield maximum biodiesel conversion and effectively enrich EPA.

## Results and discussion

### Lipid characteristics of *Nannochloropsis* sp. biomass

In this study, the selected *Nannochloropsis* sp. biomass had 16.48% TFAs (Table 1). It is well known that chemical composition of microalgae was greatly dependent on the microalgae species, cultivation modes, and environmental factors [9, 26, 27]. Doan and Obbard cultivated *Nannochloropsis* sp. cells in a photo-incubator using *f*/2 Guillard medium for 10 days and obtained the TFAs’ content 12% [28]. In the study of Lee and Han [29], 18.94% TFAs of *Nannochloropsis salina* were detected in an outdoor raceway pond. Moreover, as shown in Table 1, the NLs and PLs percentages of microalgal TFAs were 48.74 and 51.26%, respectively. These results agreed with the previous studies [4, 7]. For example, Ma et al. [4] analyzed the lipid composition of nine *Nannochloropsis* species, and found that their NLs’ contents were from 32.41 to 59.93% and the PLs’ contents were in the range of 36.10–57.42%.

**Table 1:** The composition of TFAs, lipid class, and fatty acid of undisrupted and disrupted microalgae biomass

	Microalgae biomass	Disrupted microalgae biomass
*A (TFAs and lipid class content)*		
TFAs (% of biomass)	16.48 ± 1.60	20.67 ± 1.26
NLs (% of TFAs)	48.74 ± 2.72	46.58 ± 2.80
PLs (% of TFAs)	51.26 ± 2.07	53.42 ± 1.94

	TFAs	NLs	PLs	TFAs	NLs	PLs
*B (Fatty acid composition)*						
C14:0	5.33 ± 0.40	4.42 ± 0.35	6.13 ± 0.70	5.79 ± 0.14	4.26 ± 0.32	6.62 ± 0.47
C16:0	23.30 ± 1.53	26.23 ± 1.59	16.39 ± 1.03	22.28 ± 1.41	25.44 ± 1.55	17.03 ± 1.42
C16:1	20.67 ± 1.97	22.34 ± 1.24	16.92 ± 1.32	18.39 ± 1.23	20.38 ± 0.96	15.91 ± 0.89
C18:0	2.12 ± 0.06	0.51 ± 0.08	2.41 ± 0.27	1.98 ± 0.28	0.50 ± 0.04	2.87 ± 0.30
C18:1	1.44 ± 0.11	1.57 ± 0.21	1.16 ± 0.18	1.71 ± 0.05	1.88 ± 0.24	1.07 ± 0.10
C18:2	0.76 ± 0.18	0.64 ± 0.10	0.68 ± 0.08	0.31 ± 0.01	0.74 ± 0.13	0.49 ± 0.03
C18:3	0.58 ± 0.04	0.68 ± 0.06	0.54 ± 0.06	0.62 ± 0.06	0.77 ± 0.09	0.50 ± 0.05
C20:4n-6	5.46 ± 0.38	6.42 ± 0.57	3.26 ± 0.43	5.76 ± 0.35	7.62 ± 0.48	2.26 ± 0.18
C20:5n-3	40.34 ± 2.13	37.19 ± 1.70	52.51 ± 1.98	43.18 ± 2.84	38.41 ± 1.86	53.25 ± 2.19
∑ SFAs^a^	30.75 ± 1.27	31.16 ± 1.82	24.93 ± 1.46	30.05 ± 1.06	30.20 ± 1.65	26.52 ± 0.92
∑ MFAs^b^	22.11 ± 1.33	23.91 ± 1.76	18.08 ± 1.31	20.10 ± 1.49	22.26 ± 1.39	16.98 ± 1.03
∑ PUFAs^c^	47.14 ± 2.43	44.93 ± 2.04	56.99 ± 1.74	49.87 ± 2.71	47.54 ± 2.10	56.50 ± 1.97

^a^SFAs contained C_14:0_, C_16:0_, and C_18:0_

^b^MFAs contained C_14:1_, C_16:1_, and C_18:1_

^c^PUFAs contained C_18:2_, C_18:3_, C_20:4_, and C_20:5_

Furthermore, the fatty acid composition of TFAs, NLs, and PLs of *Nannochloropsis* sp. biomass was determined (Table 1). NLs fraction had 26.23% PA (palmitic acid, C_16:0_), 22.34% POA (palmitoleic acid, C_16:1_), 6.42% AA (arachidonic acid, C_20:4n−6_), 37.19% EPA, 31.16% SFAs (saturated fatty acids), 23.91% MFAs (monounsaturated fatty acids) and 44.93% PUFAs, while these indexes of PLs fraction were 16.39, 16.92, 3.26, 52.51, 24.93, 18.08, and 56.99%, respectively. The previous works showed that PLs’ fraction of *Nannochloropsis* species usually gave higher EPA and PUFAs’ contents compared with NLs fraction [8, 9, 15]. For the TFAs of *Nannochloropsis* sp. biomass, EPA accounted for 40.34%; which was comparable [7] and higher than reported data [4–6, 8]. These results in Table 1 indicated that the *Nannochloropsis* sp. biomass selected in this study could be the promising EPA source. Thus, the following work should focus on developing a green and efficient process and then optimizing its parameters for selectively converting SFAs and MFAs into biodiesel and effectively concentrating EPA into NPLs fraction (NLs and PLs).

### Screening the suitable enzyme for ethanolysis of *Nannochloropsis* sp. biomass

**Fig. 1:**
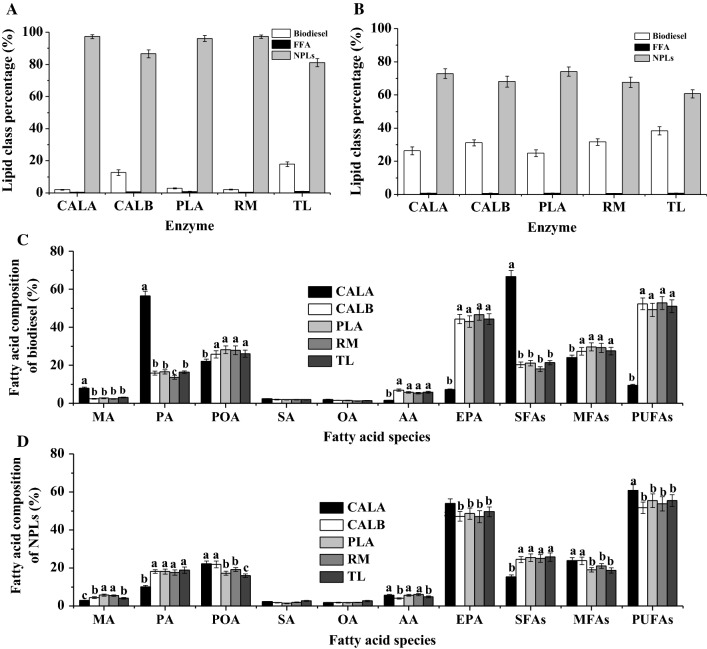
Effects of five enzymes on the lipid class percentage. **A** Undisrupted algal biomass; **B** disrupted algal biomass, and fatty acid composition of biodiesel (**C**) and NPLs (**D**) in the enzymatic ethanolysis of undisrupted and disrupted microalgae biomass. Reaction conditions: microalgae biomass (1 g dry weight), ethanol/biomass ratio (v/w) 10:1, reaction temperature 35 °C, water content 10% based on the microalgae biomass and ethanol, enzyme weight 10% based on the microalgae biomass, and reaction time 24 h. ^a,b,c^The mean values in the same line were significantly different (*p* < 0.05)

Figure 1A shows the biodiesel conversion of five enzymes using the undisrupted microalgae biomass. After 24 h of ethanolysis, biodiesel conversions of CALA, CALB, PLA, RM, and TL when using original microalgae biomass were 2.09, 12.72, 2.98, 2.20, and 17.93%, respectively, which were significantly lower than the results of López et al. [5]. One of the possible reasons was that cell wall of microalgae impeded the interaction between intracellular lipids and enzyme [5, 30]. To address this bottleneck for higher biodiesel conversion, cellulase pretreatment on *Nannochloropsis* sp. biomass was proved to be a mild, cost-effective, and efficient cell disruption method [31–34] and carried out in this study. After hydrolysis, the disrupted microalgae biomass achieved 20.67% TFAs which was higher than that of undisrupted biomass (Table 1), since partial carbohydrates of microalgal cell wall were hydrolyzed by cellulase. It was worth noting that enzymatic disruption by cellulase for *Nannochloropsis* sp. biomass did not affect the percentages of NLs and PLs, or the fatty acid composition of TFAs, NLs, and PLs in comparison to the undisrupted algal biomass (Table 1). In this case, it was necessary to re-evaluate the biodiesel conversions of these five enzymes by ethanolysis of the disrupted *Nannochloropsis* sp. biomass.

As expected, the biodiesel conversion of cellulase disrupted *Nannochloropsis* sp. biomass distinctly increased to 26.40% for CALA, 31.23% for CALB, 25.01% for PLA, 31.72% for RM, and 38.46% for TL (Fig. 1B). These results further demonstrated that cellulase positively disrupted the cell wall of *Nannochloropsis* sp. to release intercellular lipids for biodiesel production during ethanolysis [5, 6]. Moreover, as shown in Fig. 1A, B, FFA contents were very low (< 1%) after the ethanolysis of microalgae biomass with lipases/phospholipase, which was consistent with our previous study using fish oil [21]. In this case, the main lipidic class in enzymatic ethanolysis could be regarded as biodiesel and NPLs.

Fatty acid compositions of biodiesel and NPLs by five enzymes were important indexes to evaluate the ability of EPA and PUFAs enrichment. Figure 1C, D only presents the results with disrupted *Nannochloropsis* sp. biomass because of low biodiesel conversions for unpretreated groups. It was clear that CALA produced the biodiesel with the highest MA (myristic acid, C_14:0_, 7.91%), PA (56.43%), and SFAs (66.64%) contents, and the lowest AA (1.55%), EPA (7.13%), and PUFAs (9.35%) contents compared to CALB, PLA, RM, and TL (Fig. 1B). Besides, CALA gave the lowest MA (2.88%), PA (10.05%), and SFAs (15.29%) contents, and the highest EPA (53.99%) and PUFAs (60.80%) contents of NPLs in comparison to the other four enzymes (Fig. 1D). These results were consistent with our recent work, reporting that CALA exhibited strong substrate selectivity towards EPA in the ethanolysis of fish oil [21]. The catalytic substrate selectivity of lipase or phospholipase was associated with its lid covering active-site structure [16–18, 24]. CALB, PLA, RM, and TL had a short lid possessing 5 (residues 142–146) [20], 12 (residues 245–256) [35], 15 (residues 82–96) [18], and 8 (residues 86–93) [17] amino acids, respectively. Differently, CALA owned a unique and long lid structure with 92 amino acid residues (217–308) [16, 36]. During interfacial activation, some key charged residues (e.g., Glu, Arg, and His) of CALA’s lid might form an active-site flap to preferentially accommodate the SFAs acyl groups of substrate for FAEEs generation in the ethanolysis [16, 36]. To further improve biodiesel conversion and increase EPA content of NPLs, the reaction parameters of enzymatic ethanolysis were optimized with the suitable enzyme (CALA).

### Effects of ethanol-to-biomass ratio

In the enzymatic ethanolysis, it is important to determine ethanol concentration to obtain maximum biodiesel and simultaneously highest enrichment of EPA in the products, since ethanol acts as both substrate and reaction medium [21, 23, 37]. As observed in many studies, the change of ethanol concentration alters the polarity of the reaction system and the activity coefficient of water, and impacts the shifting of reaction equilibrium, thus affecting the conversions of oil substrate and the catalytic specificity of enzyme [10, 21, 24].

**Fig. 2:**
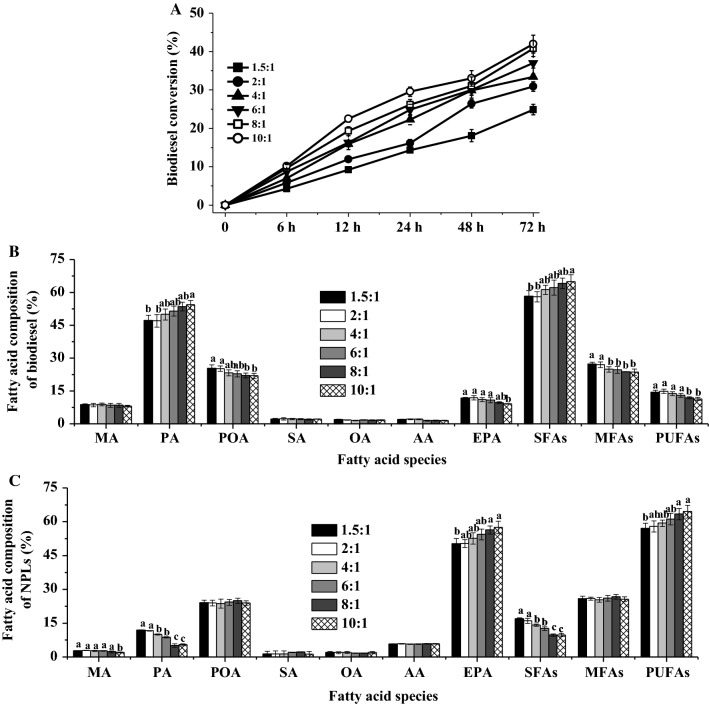
Effects of different ethanol/biomass ratio (v/w) on the biodiesel conversion (**A**), and fatty acid composition of biodiesel (**B**) and NPLs’ fractions (**C**) by the enzymatic ethanolysis with CALA. Reaction conditions: microalgae biomass (1 g dry weight), ethanol/biomass ratio (v/w) 1.5–10:1, reaction temperature 35 °C, water content 10%, enzyme weight 10%, and reaction time 72 h. ^a,b,c^The mean values in the same line were significantly different (*p* < 0.05)

The ethanol/biomass ratios were selected in the range of 1.5:1–10:1 due to the fact that lower ratios could not homogenize microalgae biomass, ethanol, and enzyme in the reaction system. Figure 2A shows how the biodiesel conversion changed with different ratios of ethanol to biomass as time evolved. At the ratio of 1.5:1, CALA gave the biodiesel conversion of 24.88% after 72 h of ethanolysis. With higher ethanol/biomass ratios from 1.5:1 to 8:1, the biodiesel conversion of CALA gradually increased. This could be that higher ethanol concentration promoted the solubility of intercellular lipids and shifted reaction equilibrium to generate biodiesel [5]. However, when the ethanol/biomass ratio was further increased to 10:1, CALA cannot increase the biodiesel conversion (41.94%) any further compared to 8:1 ethanol/biomass ratio (40.79%). To reduce the ethanol utilization, the ethanol/biomass ratio of 8:1 was chosen.

The fatty acid compositions of biodiesel and NPLs by CALA at different ethanol/biomass ratios are shown in Fig. 2B, C. With respect to the biodiesel, an increase in the ethanol/biomass ratio led to the increases in PA and SFAs’ contents and gradual decreases in EPA and PUFAs’ contents. Meantime, as ethanol concentration increased, the PA and SFAs’ contents of NPLs decreased, while EPA and PUFAs’ contents of NPLs increased. However, changing ethanol concentration did not significantly influence the POA and MFAs’ contents of NPLs. In fact, POA and MFAs’ contents of NPLs were related with biodiesel conversion and their contents in biodiesel (POA or MFAs’ content of NPLs = POA or MFAs’ content of TFAs–POA or MFAs’ content of biodiesel × biodiesel conversion). These results suggested that CALA did not exhibit substrate selectivity towards POA. Nevertheless, this enzyme performed strong ability to discriminate against EPA in high ethanol concentration (≥ 8:1), which may be because high ethanol concentration might interact with some aromatic residues of lid structure to form a smaller tunnel-like-binding site where CALA cannot accommodate the EPA moieties during interfacial activation [16, 36].

### Effects of reaction temperature

In a given enzymatic reaction, the optimal reaction temperature improved substrate solubility, enzyme thermostability, and catalytic activity, as well as the affinity between substrates and enzymes [10, 21, 24, 38]. In the previous experiments, suitable temperatures of CALA were in the range 20–60 °C [21, 36]. Thus, the effects of reaction temperatures (25, 35, 45, and 55 °C) on the biodiesel conversion and fatty acid composition were investigated.

**Fig. 3:**
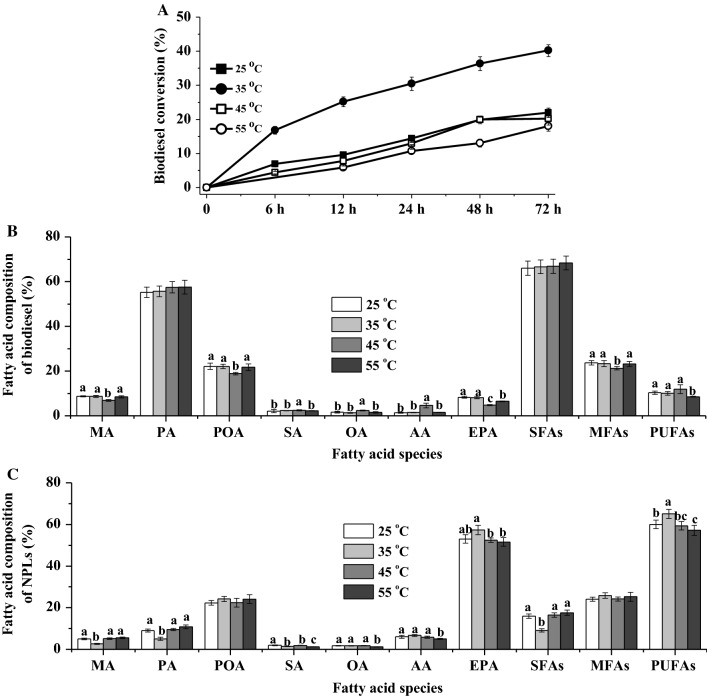
Effects of different reaction temperature on the biodiesel conversion (**A**), and fatty acid composition of biodiesel (**B**) and NPLs’ fractions (**C**) by the enzymatic ethanolysis with CALA. Reaction conditions: microalgae biomass (1 g dry weight), ethanol/biomass ratio (v/w) 8:1, reaction temperature 25–55 °C, water content 10%, enzyme weight 10%, and reaction time 72 h. ^a,b,c^The mean values in the same line were significantly different (*p* < 0.05)

The effect of reaction temperature (25–55 °C) on biodiesel conversion of CALA is shown in Fig. 3A. Obviously, the highest biodiesel conversion (40.23%) was obtained by CALA at 35 °C, which was in line with our previous study using fish oil. Lower reaction temperature (25 °C) could have a negative impact on substrate solubility, leading to the decrease in biodiesel conversion [10, 17, 18, 24]. Other than that, elevated reaction temperature (45 and 55 °C) affected the enzyme thermostability and reduced catalytic activity to a great extent [10, 17, 18, 24, 38], resulting in the low biodiesel conversion (Fig. 3A).

Figure 3B, C describes the fatty acid composition of biodiesel and NPLs by CALA after 72 h of ethanolysis. It was not surprising that CALA produced NPLs containing the lowest contents of MA (2.66%), PA (5.04%), and SFAs (9.09%) at 35 °C, while the highest MA, PA, and SFAs yield (biodiesel conversion × specific fatty acid content of biodiesel) of microalgae TFAs. In addition, the highest EPA (57.31%) and PUFAs (65.05%) contents of NPLs were produced by CALA at 35 °C. The phenomenon might be that CALA maintained its optimal conformational changes and sufficient flexibility of lid structure in optimal reaction temperature in which the substrate-binding tunnel structure of enzyme reacted with microalgal lipids containing less twisting acyl groups (e.g., PA) for FAEEs’ generation and enriched EPA [16]. It was, therefore, preferable to operate at 35° as optimal temperature with respect to achieving the highest biodiesel conversion and the greatest efficacy for EPA enrichment.

### Effects of water content

**Fig. 4:**
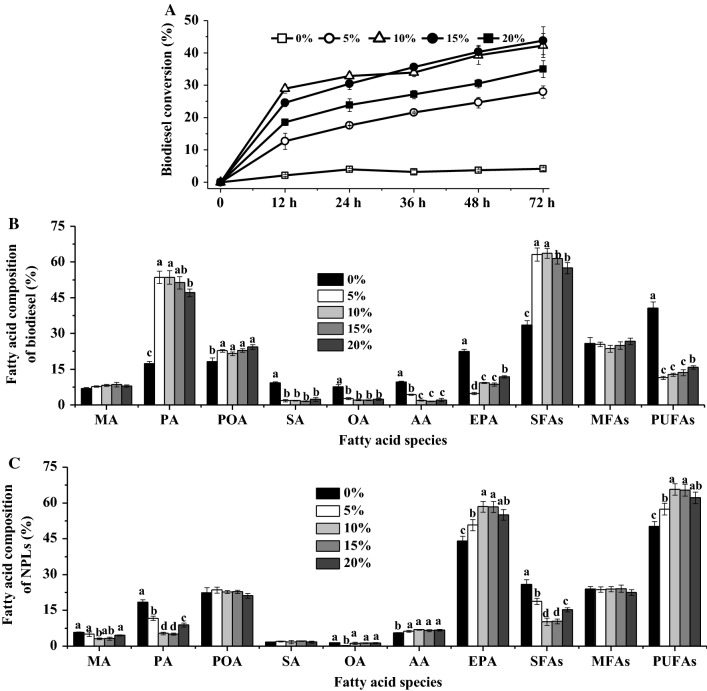
Effects of water content on the biodiesel conversion (**A**), and fatty acid composition of biodiesel (**B**) and NPLs’ fractions (**C**) by the enzymatic ethanolysis with CALA. Reaction conditions: microalgae biomass (1 g dry weight), ethanol/biomass ratio (v/w) 8:1, reaction temperature 35 °C, water content 0–20%, enzyme weight 10%, and reaction time 72 h. ^a,b,c,d^The mean values in the same line were significantly different (*p* < 0.05)

The effects of varying water content on the biodiesel conversion of CALA were investigated (Fig. 4A). As reaction time progresses, CALA performed a low biodiesel conversion (< 5%) in the anhydrous condition, which agreed well with our previous study [39]. In the 5% water content, CALA yielded the biodiesel conversion of 27.93%. When the water content was in the range of 10–15%, the biodiesel conversion increased to 42.31–43.82%. However, high water content (20%) in the ethanolysis reaction remarkably decreased the biodiesel conversion (35.01%). Available evidences pointed out that optimal water content greatly increased the catalytic activity at the lipid/water interface in the lipase-catalyzed reaction with organic solvent(s) [10, 24, 37, 40].

Figure 4B, C shows the results about the fatty acid composition of biodiesel and NPLs in different water contents. When water content was increased from 0 to 10%, MA and PA and SFAs’ contents of biodiesel gradually increased, while MA, PA, and SFAs’ contents of NPLs significantly decreased. Moreover, there were no significant differences in the contents of MA, PA, EPA, SFAs, and PUFAs of NPLs between the water content of 10 and 15%. Nevertheless, a decrease in the EPA and PUFAs’ contents of NPLs was observed in 20% water content. In general, during interfacial activation or conformational change, the optimal water activity kept the three-dimensional structure of CALA with a larger loop/helix/loop fragment covering the acyl-binding site [16]; in this case, the enzyme might preferentially catalyze the short lipid tails, namely SFAs acyl moieties of lipid substrate [16, 36]. Thus, to yield the highest biodiesel conversion and effectively enrich EPA into NPLs, the optimal water content was 10–15% for CALA in the ethanolysis of disrupted *Nannochloropsis* sp. biomass.

### Effects of enzyme (CALA) weight

**Fig. 5:**
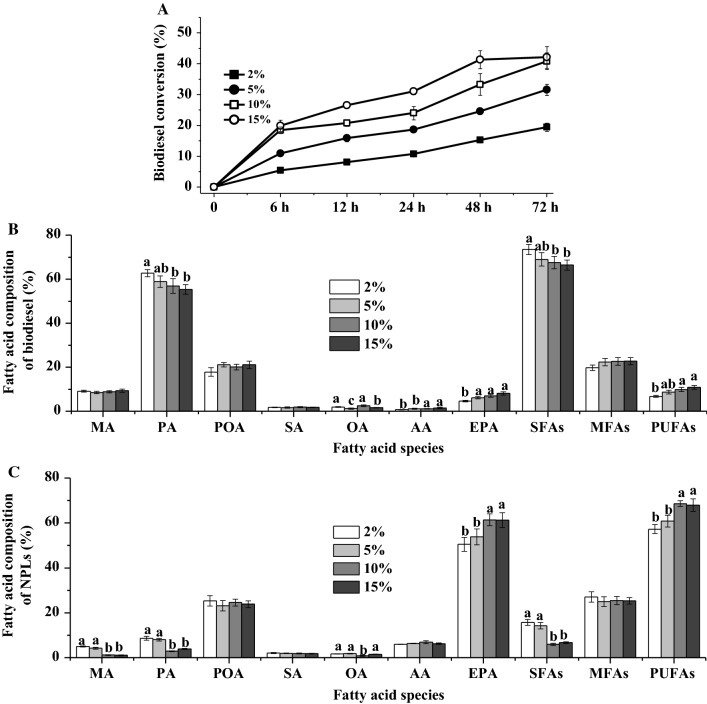
Effects of lipase weight on the biodiesel conversion (**A**), and fatty acid composition of biodiesel (**B**) and NPLs fractions (**C**) by the enzymatic ethanolysis with CALA. Reaction conditions: microalgae biomass (1 g dry weight), ethanol/biomass ratio (v/w) 8:1, reaction temperature 35 °C, water content 10%, enzyme weight 2–15%, and reaction time 72 h. ^a,b^The mean values in the same line were significantly different (*p* < 0.05)

The effects of varying enzyme weights (2, 5, 10, and 15%, based on microalgae biomass, wt%) on ethanolysis catalyzed by CALA are shown in Fig. 5. Figure 5A displays the results about how the biodiesel conversion of CALA was affected. An increase in lipase weight from 2 to 5% led to a significant increase in the biodiesel conversion from 19.45 to 40.84%. However, no significant change in the biodiesel conversion was observed at the lipase weight of 10 and 15%. Moreover, high lipase weight could short the reaction time to yield the same biodiesel conversion. For instance, CALA attained similar biodiesel conversions at 10% lipase weight in 72 h (40.84%), while at 15% lipase weight only in 48 h (41.35%). Undoubtedly, the time–space efficiency of this process should be re-assessed to produce biodiesel for scale-up application.

At the end of trials, the fatty acid compositions of biodiesel and NPLs are determined and presented in Fig. 5B, C. CALA produced biodiesel with the highest PA and SFAs’ contents and the lowest EPA and PUFAs’ contents at 5% lipase weight, ascribing to its fatty acid selectivity mainly catalyzing SFAs of lipids before the reaction equilibrium. As for NPLs fraction, MA, PA, and SFAs’ contents significantly decreased and EPA and PUFAs’ contents sharply increased as increasing lipase weight from 2 to 10%. Furthermore, there was no significant change in the fatty acid composition of NPLs at the lipase weight of 10 and 15%, indicating a level-off effect. From an economic consideration, 10% enzyme weight was selected in this study.

### Effects of reaction time

**Fig. 6:**
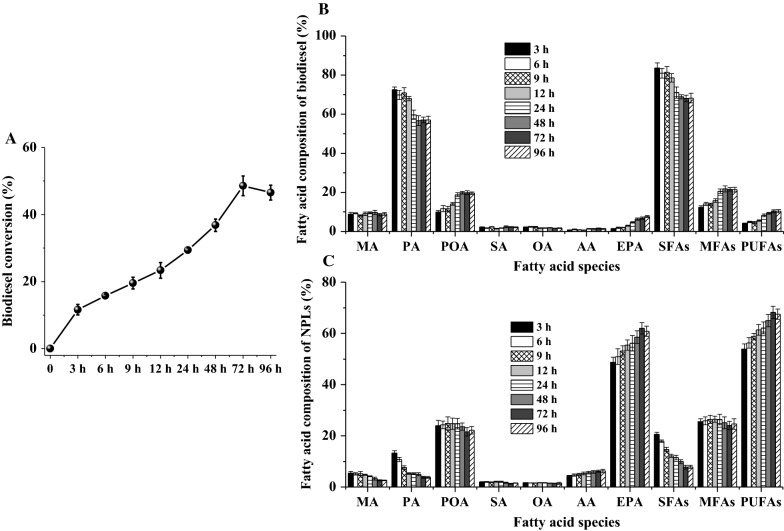
Changes of biodiesel conversion (**A**), fatty acid composition of biodiesel (**B**) and NPLs (**C**) fractions against reaction time during enzymatic ethanolysis with CALA. Reaction conditions: microalgae biomass (1 g dry weight), ethanol/biomass ratio (v/w) 8:1, reaction temperature 35 °C, water content 10%, enzyme weight 10%, and reaction time 96 h

Figure 6 depicts the changes of the biodiesel conversion, and the fatty acid composition of biodiesel and NPLs along with reaction progress in the ethanolysis of *Nannochloropsis* sp. biomass. Results in Fig. 6A revealed that the biodiesel conversions continuously increased to a maximum (46.53–48.57%) until 72 h and then started to decrease. In this study, the biodiesel conversion of CALA was lower than that of the results of López et al. [5] and Law et al. [6] because of the differences in microalgae species, sources of biocatalyst, and alcohol types.

Figure 6B, C shows the changes of individual fatty acid contents of biodiesel and NPLs as the reaction progressed. In the initial 12 h, CALA produced the biodiesel containing high contents of PA (68.02–72.51%) and SFAs (78.57–83.67%); in this case, high biodiesel conversion decreased the PA and SFAs’ contents of NPLs fraction. It was noted that POA content of biodiesel increased first (3–24 h, from 9.97 to 18.83%) and then kept constant afterwards (19.59–19.89%). As discussed above, CALA tended to selectively catalyze SFAs of lipids for FAEEs, namely PA > POA. Furthermore, even though microalgal TFAs had a higher EPA content than PA (Table 1), the EPA content (1.43–7.63%) of biodiesel was significantly lower than that of POA. The result suggested that CALA preferentially transesterified POA of lipid for biodiesel in comparison to EPA, due to the fact that EPA had longer carbon chain length (C_20_) and more number of double bonds (5). Thus, the order of fatty acid selectivity for CALA was PA > POA > EPA.

Moreover, after the ethanolysis of the disrupted *Nannochloropsis* sp. biomass with CALA, PA, and SFAs’ contents of NPLs decreased by 84.05 and 74.34%, respectively, compared with microalgae TFAs, while EPA (60.81%) and PUFAs (67.49%) contents of NPLs were 1.51- and 1.43-fold of those in original microalgal TFAs. To the best of our knowledge, the obtained EPA content of *Nannochloropsis* sp. NPLs was significantly higher than the results of our recent work (50.86% in MAGs) and other author’s studies (33.8–40% in glycerides) about EPA enrichment by ethanolysis or hydrolysis of fish oil [21–23, 37, 41–43]. These results showed that this process via enzymatic ethanolysis of the disrupted *Nannochloropsis* sp. biomass with CALA was a simple and efficient approach for enrich EPA into NPLs along with biodiesel production.

### Evaluation of two different *Nannochloropsis* species by the enzymatic ethanolysis for biodiesel and EPA enrichment

To verify whether CALA could exhibit superior performance for different sources of *Nannochloropsis* species biomass, two *Nannochloropsis* species (IMET1 and Salina 537) were evaluated. After hydrolysis by cellulase, the lipid characteristics of IMET1 and Salina 537 are examined and presented in Table 2. The TFAs’ contents of IMET1 and Salina 537 were 14.61 and 18.02%, respectively. IMET1 had 60.23% NLs and 39.77% PLs, while Salina 537 had 51.28% NLs and 48.72% PLs (Table 2). In view of the fatty acid composition, IMET1 TFAs had 27.46% PA, 31.63% POA, 8.47% oleic acid (OA, C_18:1_), 18.70% EPA, 33.18% SFAs, 40.10% MFAs, and 26.35% PUFAs. The main fatty acids of Salina 537 TFAs were PA (31.34%), POA (26.75%), and EPA (24.50%). The previous studies stated that different *Nannochloropsis* species produce lipids with distinct fatty acid composition, because of the differences in microalgae species and their individual lipid metabolism [4, 15, 44].

**Table 2:** Lipid characteristics and fatty acid composition of TFAs, biodiesel, and NPLs after the ethanolysis with CALA using the two disrupted *Nannochloropsis* species (IMET1 and Salina 537) biomass

	IMET1	Salina 537
*A (Lipid characteristics)*		
TFAs (% of biomass)	14.61 ± 1.41	18.02 ± 1.52
NLs (% of TFAs)	60.23 ± 2.13	51.28 ± 2.47
PLs (% of TFAs)	39.77 ± 1.70	48.72 ± 2.66

	TFAs^a^	Biodiesel^b^	NPLs^b^	TFAs^a^	Biodiesel^b^	NPLs^b^
*B (Fatty acid composition)*						
C14:0	4.70 ± 0.11	5.57 ± 0.21	1.42 ± 0.15	6.08 ± 0.54	7.64 ± 0.58	1.22 ± 0.33
C16:0	27.46 ± 1.23	52.94 ± 2.73	4.26 ± 0.22	31.34 ± 1.29	58.60 ± 2.64	5.62 ± 0.47
C16:1	31.63 ± 2.28	20.55 ± 1.45	33.28 ± 3.49	26.75 ± 1.52	18.88 ± 0.47	29.03 ± 1.55
C18:0	1.02 ± 0.04	0.90 ± 0.10	0.87 ± 0.16	1.23 ± 0.04	1.03 ± 0.08	1.48 ± 0.16
C18:1	8.47 ± 0.83	10.59 ± 0.81	5.20 ± 1.00	4.29 ± 0.00	5.19 ± 0.33	3.22 ± 0.40
C18:2	3.20 ± 0.31	2.89 ± 0.18	1.85 ± 0.28	2.53 ± 0.16	2.82 ± 0.18	2.19 ± 0.19
C18:3	2.40 ± 0.02	2.43 ± 0.20	1.38 ± 0.23	0.69 ± 0.15	1.15 ± 0.11	0.14 ± 0.04
C20:4n-6	2.05 ± 0.07	0.82 ± 0.03	2.46 ± 0.13	2.62 ± 0.12	1.33 ± 0.19	3.16 ± 0.26
C20:5n-3	18.70 ± 0.51	1.86 ± 0.16	50.06 ± 2.82	24.50 ± 0.62	3.20 ± 0.33	53.73 ± 3.40
∑ SFAs	33.18 ± 1.13	60.41 ± 2.85	6.55 ± 0.41	38.65 ± 2.48	67.27 ± 1.75	8.32 ± 0.89
∑ MFAs	40.10 ± 2.55	27.14 ± 1.46	37.48 ± 2.59	31.04 ± 2.09	24.07 ± 0.80	32.25 ± 1.95
∑ PUFAs	26.35 ± 0.71	8.00 ± 0.39	55.75 ± 2.10	30.34 ± 1.28	8.50 ± 0.49	59.22 ± 2.58

^a^The fatty acid composition was determined by GC–MS using the hydrolyzed *Nannochloropsis* biomass

^b^The fatty acid composition of biodiesel and NPLs was determined by GC-MS after enzymatic ethanolysis with CALA under the optimal conditions

**Fig. 7:**
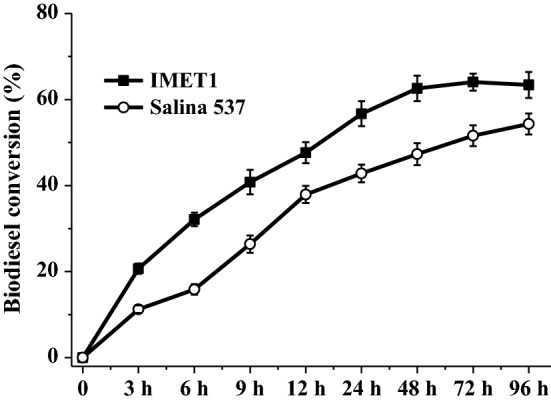
The biodiesel conversions of CALA by enzymatic ethanolysis of two *Nannochloropsis* species (IMET1 and Salina 537) biomass under the optimal reaction conditions. Reaction conditions: microalgae biomass (1 g dry weight), ethanol/biomass ratio (v/w) 8:1, reaction temperature 35 °C, water content 10%, enzyme weight 10%, and reaction time 96 h

In the enzymatic ethanolysis of microalgae biomass with CALA under the optimal reaction conditions, the biodiesel conversions of IMET1 and Salina 537 gradually increased in the initial 48 h (Fig. 7). At the end of trials, the biodiesel conversions were 63.41% for IMET1 and 54.33% for Salina 537 (Fig. 7). It should be noted that CALA catalyzed the disrupted IMET1 biomass to achieve a higher biodiesel conversion compared with the result of Salina 537 (Fig. 7). The possible reason was that IMET1 had a higher level of NLs (Table 2). It has been reported that higher PLs’ content distinctly decreased biodiesel conversion in the lipase-catalyzed transesterification [45, 46].

Moreover, the fatty acid compositions of biodiesel and NPLs obtained by CALA are presented in Table 2. Clearly, biodiesels obtained by CALA with IMET1 and Salina 537 biomass had higher contents of PA (52.94–58.60%), POA (18.88–20.55%), SFAs (60.41–67.27%), and MFAs (24.07–30.14%), and lower contents of EPA (1.86–3.20%) and PUFAs (8–8.50%), agreeing with the trends of CALA using *Nannochloropsis* sp. biomass (Fig. 6). On the other hand, after 96 h of ethanolysis with IMET1 and Salina 537 biomasses, microalgal NPLs mainly had POA (29.03–33.28%) and EPA (50.06–53.73%), as shown in Table 2. These results indicated that different compositions in lipidic class and fatty acids influenced the fatty acid selectivity of lipase, as previously described [42, 45–49]. Although the EPA values of NPLs via enzymatic ethanolysis of IMET1 and Salina 537 biomasses were a bit lower than the one of *Nannochloropsis* sp. biomass (Fig. 6), these data in this study were comparable and even higher than the previous reported ones [21–23, 37, 41–43].

In addition, Table 2 shows that the EPA contents of microalgal NPLs in IMET1 and Salina 537 produced by CALA were 1.68 and 1.19 times more than the one in their original TFAs. These results further showed that CALA with the unique fatty acid selectivity could be a promising biocatalyst to catalyze the *Nannochloropsis* biomass for biodiesel production and EPA enrichment. To further improve the EPA content of microalgal NPLs, the CALA structure needs to be modified for preferentially transesterifying PA and POA of *Nannochloropsis* lipids by a series of biotechnologies [34].

## Conclusions

For the first time, a simple and efficient process was successfully implemented to highly concentrate EPA using *Nannochloropsis* biomass via enzymatic ethanolysis with liquid CALA along with biodiesel production. Results showed that enzymatic cell disruption pretreatment on *Nannochloropsis* biomass significantly increased the biodiesel conversion of CALA. Under the optimized reaction conditions, the highest biodiesel conversion of CALA was 46.53–63.41%. For three different *Nannochloropsis* species, the EPA content in NPLs fraction was enriched to 50.06–60.81%, 1.51-fold for *Nannochloropsis* sp. biomass, 2.68-fold for IMET1, and 2.19-fold for Salina 537 above the initial levels, respectively. In all, this study provides a novel and potential strategy of *Nannochloropsis* biomass for nutraceutical EPA enrichment and sustainable biodiesel production to improve economic feasibility.

## Materials and methods

### Enzymes and microalgae

In this study, all solvents and reagents purchased from Sinopharm Chemical Reagent Co., Ltd (Beijing, China) were of chromatographic or analytic grade. Cellulase from *Trichoderma viride* (15,000 U/g) was obtained from Sinopharm Chemical Reagent Co., Ltd (Beijing, China). CALA, CALB, PLA, RM, and TL were purchased from Novo Nordisk A/S Bagsvaerd, Denmark (Table 3). One unit of cellulase activity was equal to the amount of enzyme which liberated 1.0 μg glucose from cellulose substrate in 1 min at 50 °C and pH 4.8. One KLU of lipase/phospholipase was defined as the amount of enzyme that liberated 1 mmol/min of titratable butyric acid from tributyrin in pH 7.2 at 37 °C. Microalgae powder of *Nannochloropsis* sp. was purchased from Yantai Hairong Biology Technology Co., Ltd (Shandong, China) and stored in the dark − 20 °C.

**Table 3:** The details of five lipases/phospholipase used in this study

Enzyme	Microorganism	Enzyme activity	Commercial name	Abbreviation
Lipase A	*Candida antarctica*	6 KLU/g	NovoCor^®^ AD L	CALA
Lipase B	*Candida antarctica*	5 KLU/g	Lipozyme^®^ CALB L	CALB
Phospholipase	Genetically *Aspergillus oryzae*	10 KLU/g	Lecitase^®^ Ultra phospholipase A1	PLA
Lipase RM	*Rhizomucor miehei*	20 KLU/g	Palatase 20000L	RM
Lipase TL	*Thermomyces lanuginosus*	100 KLU/g	Lipozyme^®^ TL 100L	TL

*Nannochloropsis oceanica* IMET1 (named as IMET1) was from the Institute of Marine and Environmental Technology, the University of Maryland (IMET, USA). *Nannochloropsis salina* CCMP 537 (named as Salina 537) was from the National Center for Marine Algae and Microbiota (NCMA, USA). Microalgal cells for these two species were grown in 800 mL glass columns illuminated with 60 μE m^−2^ s^−1^ and aerated 2.5% CO_2_ enrich air using the modified *f*/2 medium of 600 mL. The modified f/2 medium [44, 50] was composed of (mg L^−1^): NaNO_3_, 750; NaH_2_PO_4_·H_2_O, 48; Na_2_EDTA, 4.36; FeCl_3_·6H_2_O, 3.16; CuSO_4_·5H_2_O, 0.01; ZnSO_4_·7H_2_O, 0.025; CoCl_2_·6H_2_O, 0.012; MnCl_2_·4H_2_O, 0.18; Na_2_MoO_4_·2H_2_O 7.0 × 10^−3^; Vitamin B_1_, 0.1; Vitamin B_12_, 1.0 × 10^−3^; Vitamin H, 1.0 × 10^−3^. The pH and salinity of medium were set to 7.8 and 25 g L^−1^, respectively. After 10 days of cultivation, the microalgal cells were centrifuged at 5000 rpm for 10 min. The microalgae paste (water content, around 80%) was washed with distilled water for three times. The precipitate was collected, lyophilized in freeze-drier, and stored at − 20 °C for further study.

### Pretreatment of *Nannochloropsis* sp. biomass by cellulase

Cellulase was utilized to disrupt *Nannochloropsis* sp. biomass according to a modified method of Wu et al. [31]. The hydrolysis mixtures consisted of 100 g *Nannochloropsis* sp. powder, 20,000 U cellulase, and 2 L 0.1 M phosphate buffer solution (pH 4). The hydrolysis reaction was conducted at 50 °C for 12 h in a water bath shaker (MQS-30S, China). After the enzymatic hydrolysis, the pretreated microalgae biomass was collected by centrifugation at 5000 rpm for 5 min. The precipitate was collected, lyophilized in freeze-drier, and stored at − 20 °C.

### Enzymatic ethanolysis of the disrupted microalgae biomass

First, the best enzyme (CALA, CALB, TL, RM, and PLA) was screen to produce biodiesel and enrich EPA by ethanolysis of the pretreated *Nannochloropsis* sp. biomass. Dark-colored and screw-capped flasks (25 mL) containing the pretreated microalgae (1 g dry biomass), anhydrous ethanol (10 mL), distilled water (0.78 g), and lipase/phospholipase (0.1 mL) were incubated in water bath shaker (MQS-30S, China). After ethanolysis of 24 h, the final products were analyzed by a gas chromatography–mass spectrometry (GC–MS).

To further improve biodiesel conversion and EPA enrichment by the suitable enzyme, the reaction parameters such as anhydrous ethanol-to-dry microalgae biomass ratio (v/w, 1.5–10:1), reaction temperature (25–55  °C), water content based on dry biomass and ethanol (0–20%), enzyme weight based on dry biomass (2–15%), and reaction time (0–96 h) were investigated. During the ethanolysis, samples were withdrawn periodically for gas chromatography–mass spectrometer (GC–MS) analysis.

### Determination of total fatty acids (TFAs) by GC–MS

Based on our previous method [39], 10 mg microalgae biomass was methylated to quantify the TFAs’ content using heptadecanoic acid methyl ester (HAME, 0.1 mg/mL) as an internal standard. Fatty acid methyl esters (FAMEs) were recorded by the gas chromatography–mass spectrometry (GC–MS, GC–MS–QP 2010 SE, Shimadzu, Japan) equipped with a Stabliwas-DA capillary column (30 m × 0.25 mm × 0.25 μm, Shimadzu, Japan) [39]. The initial oven temperature was set at 150 °C and subsequently increased to 180 °C at a rate of 10 °C/min and then raised to 220 °C at the speed of 2 °C/min, and finally holding for 10 min. The injector temperature was 250 °C and the injection volume was 1 μL. The fatty acid species were identified by a standard mixture of 37 FAMEs (C14:0–C22:6, Supleco Inc.):

1 $${\text{TFAs}}\,{\text{content of microalgal biomass }}\left( \% \right)\, = \,\frac{{m_{\text{TFAs}} }}{{m_{\text{Algae}} }}\, \times \,100\%$$

where *m*_TFAs_ was the TFAs by GC–MS, mg; *m*_Algae_ was the weight of the treated microalgae biomass, mg.

### Fractionation of microalgae lipids

Dried microalgae biomass (100 mg) was extracted with chloroform:methanol:distilled water (2:1:0.8, v/v/v) for three times [5, 39]. After centrifugation (5000 rpm, 5 min), the chloroform phase was collected and evaporated by nitrogen. A silica gel column (diameter, 12 mm, length, 150 mm) containing 10 g of silica gel (200–300 mesh) was used to separate and purify extracted microalgae lipids for neutral lipids (NLs) and polar lipids (PLs). The NLs’ fraction was collected by eluting 30 mL of chloroform, while PLs fraction was collected by 30 mL of methanol [5]. The organic solvents were removed by nitrogen. The TFAs of NLs or PLs sample (2 mg) was then methylated and quantified by GC–MS with HAME (0.1 mg/mL) as the internal standard:

2 $${\text{NLs }}\,{\text{percentage of microalgal TFAs }}\left( \% \right)\, = \,\frac{{m_{\text{NLs}} }}{{m_{\text{TFAs}} }}\, \times \,100\%$$

3 $${\text{PLs percentage of microalgal TFAs }}\left( \% \right)\, = \,\frac{{m_{\text{PLs}} }}{{m_{\text{TFAs}} }}\, \times \,100\%$$

where *m*_NLs_ was the TFAs of NLs by GC–MS, mg; *m*_PLs_ was the TFAs of PLs by GC–MS, mg; *m*_TFAs_ was the TFAs by GC–MS, mg.

### Determination of biodiesel conversion

Samples were centrifuged at 5000 rpm for 5 min to collect the ethanol phase. The sediment was extracted by chloroform:methanol:water (2:1:0.8, v/v/v) for three times, and the chloroform phase was collected [5, 6]. The dried samples (ethanol and chloroform phases) were combined and dissolved with 1 mL n-hexane solution (HAME, 0.1 mg). The prepared samples were examined by GC–MS. Equation (4) was used to estimate the biodiesel conversion:

4 $${\text{Biodiesel conversion }}\left( \% \right)\, = \,\frac{{{\text{FAEEs produced by a liquid enzyme }}\left( {\text{mg}} \right)}}{{{\text{TFAs of the biomass }}\left( {\text{mg}} \right)}}\, \times \,100\%$$

### Determination of fatty acid composition of NLs and PLs (NPLs) in the ethanolysis

Exception for FAEEs, the enzymatic ethanolysis system contained TAGs, free fatty acids (FFA), diacylglycerols (DAGs), monoacylglycerols (MAGs), and PLs. TAGs, DAGs, and MAGs belonged to NLs. Our previous study had shown that FFA content was very low (< 1%) during the ethanolysis of oil in the presence of excessive ethanol [21]. In this case, the products of enzymatic ethanolysis could be divided into FAEEs and NPLs’ fraction (NLs and PLs). Of course, the FFFA content was also estimated using the following equation:

5 $$\text{FFA contentof microalgal TFAs}\,\left( \% \right)=\frac{m_\text{FFa}}{m_\text{TFAs}}\times 100\% .$$

To determine the fatty acid composition of NPLs, the samples were treated by the aforementioned procedures in “Determination of biodiesel conversion” section. Then, the dried products (3 mg) were spotted on commercial silica gel GF UV-254 plates (TLC, silica gel GF UV-254, thickness 0.25 mm, 10 cm × 20 cm), and developed in a mixture of *n*-hexane:diethyl ether:formic acid (84:16:0.04, v/v/v) for 20 min [21]. Ethyl oleate, triolein, oleic acid, 1,2-diolein, 2-oleoylglycerol, and egg yolk phosphatidylcholine were used to identify the specific lipid class. The bands representing different lipid class (TAGs, FFA, DAGs, MAGs, and PLs) were visualized under 254 nm, scraped off, and mixed together. Then, NPLs’ fraction was extracted with chloroform:methanol (2:1, v/v) for three times. After solvent removal by nitrogen, fatty acid composition of NPLs was determined by GC–MS and HAME (0.2 mg/mL) was the internal standard.

### Statistical analysis

All experiments were performed in triplicates. Data were processed by Microsoft Excel 2016 and Origin 2018 (Microcal Software Inc., Northampton, MA, USA), presenting as means (*n* = 3) ± the standard deviation. One-way analyses of variance (ANOVA) were conducted between the means using significant differences (*p* < 0.05).

## References

[1] Maeda Y, Yoshino T, Matsunaga T, Matsumoto M, Tanaka T (2018). Marine microalgae for production of biofuels and chemicals. Curr Opin Biotechnol.

[2] Shuba Eyasu S, Kifle D (2018). Microalgae to biofuels: ‘Promising’ alternative and renewable energy, review. Renew Sust Energ Rev..

[3] Perin G, Bellan A, Segalla A, Meneghesso A, Alboresi A, Morosinotto T (2015). Generation of random mutants to improve light-use efficiency of *Nannochloropsis gaditana* cultures for biofuel production. Biotechnol Biofuels.

[4] Ma Y, Wang Z, Yu C, Yin Y, Zhou G (2014). Evaluation of the potential of 9 *Nannochloropsis* strains for biodiesel production. Bioresour Technol.

[5] López E, Robles Medina A, Esteban Cerdán L, González Moreno PA, Macías Sánchez MD, Molina Grima E (2016). Fatty acid methyl ester production from wet microalgal biomass by lipase-catalyzed direct transesterification. Biomass Bioenerg.

[6] Law SQK, Halim R, Scales PJ, Martin GJO (2018). Conversion and recovery of saponifiable lipids from microalgae using a nonpolar solvent via lipase-assisted extraction. Bioresour Technol.

[7] Mitra M, Patidar SK, George B, Shah F, Mishra S (2015). A euryhaline *Nannochloropsis gaditana* with potential for nutraceutical (EPA) and biodiesel production. Algal Res.

[8] Mitra M, Patidar SK, Mishra S (2015). Integrated process of two stage cultivation of *Nannochloropsis* sp. for nutraceutically valuable eicosapentaenoic acid along with biodiesel. Bioresour Technol..

[9] Wei H, Shi Y, Ma X, Pan Y, Hu H, Li Y, Luo M, Gerken H, Liu J (2017). A type-I diacylglycerol acyltransferase modulates triacylglycerol biosynthesis and fatty acid composition in the oleaginous microalga, *Nannochloropsis oceanica*. Biotechnol Biofuels..

[10] Bornscheuer UT (2018). Enzymes in lipid modification. Annu Rev Food Sci Technol..

[11] Dong Y, Xu M, Kalueff AV, Song C (2018). Dietary eicosapentaenoic acid normalizes hippocampal omega-3 and 6 polyunsaturated fatty acid profile, attenuates glial activation and regulates BDNF function in a rodent model of neuroinflammation induced by central interleukin-1beta administration. Eur J Nutr.

[12] Du L, Yang YH, Wang YM, Xue CH, Kurihara H, Takahashi K (2015). EPA-enriched phospholipids ameliorate cancer-associated cachexia mainly via inhibiting lipolysis. Food Funct..

[13] Gribble MO, Karimi R, Feingold BJ, Nyland JF, O’Hara TM, Gladyshev MI, Chen CY (2016). Mercury, selenium and fish oils in marine food webs and implications for human health. J Mar Biol Assoc UK.

[14] Mwakalapa EB, Mmochi AJ, Muller MHB, Mdegela RH, Lyche JL, Polder A (2018). Occurrence and levels of persistent organic pollutants (POPs) in farmed and wild marine fish from Tanzania. A pilot study. Chemosphere..

[15] Lenka SK, Carbonaro N, Park R, Miller SM, Thorpe I, Li Y (2016). Current advances in molecular, biochemical, and computational modeling analysis of microalgal triacylglycerol biosynthesis. Biotechnol Adv.

[16] Ericsson DJ, Kasrayan A, Johansson P, Bergfors T, Sandstrom AG, Bäckvall JE, Mowbray SL (2008). X-ray structure of *Candida antarctica* lipase A shows a novel lid structure and a likely mode of interfacial activation. J Mol Biol.

[17] Fernandez-Lafuente R (2010). Lipase from *Thermomyces lanuginosus*: uses and prospects as an industrial biocatalyst. J Mol Catal B-Enzym..

[18] Rodrigues RC, Fernandez-Lafuente R (2010). Lipase from *Rhizomucor miehei* as a biocatalyst in fats and oils modification. J Mol Catal B-Enzym..

[19] De Maria L, Vind J, Oxenboll KM, Svendsen A, Patkar S (2007). Phospholipases and their industrial applications. Appl Microbiol Biotechnol.

[20] Idris A, Bukhari A (2012). Immobilized Candida antarctica lipase B: hydration, stripping off and application in ring opening polyester synthesis. Biotechnol Adv.

[21] He Y, Li J, Kodali S, Balle T, Chen B, Guo Z (2017). Liquid lipases for enzymatic concentration of n-3 polyunsaturated fatty acids in monoacylglycerols via ethanolysis: catalytic specificity and parameterization. Bioresour Technol.

[22] Yan X, Zhao X, Ma G, Dai L, Du W, Liu D (2018). Enzymatic ethanolysis of fish oil for selective concentration of polyunsaturated fatty acids (PUFAs) with flexible production of corresponding glycerides and ethyl esters. J Chem Technol Biot..

[23] Valverde LM, Moreno PAG, Callejón MJJ, Cerdán LE, Medina AR (2013). Concentration of eicosapentaenoic acid (EPA) by selective alcoholysis catalyzed by lipases. Eur J Lipid Sci Tech..

[24] Adlercreutz P (2013). Immobilisation and application of lipases in organic media. Chem Soc Rev.

[25] Price J, Nordblad M, Martel HH, Chrabas B, Wang H, Nielsen PM, Woodley JM (2016). Scale-up of industrial biodiesel production to 40 m^3^ using a liquid lipase formulation. Biotechnol Bioeng.

[26] Liu J, Yao C, Meng Y, Cao X, Wu P, Xue S (2018). The DeltaF/Fm’-guided supply of nitrogen in culture medium facilitates sustainable production of TAG in *Nannochloropsis oceanica* IMET1. Biotechnol Biofuels.

[27] Sun H, Zhao W, Mao X, Li Y, Wu T, Chen F (2018). High-value biomass from microalgae production platforms: strategies and progress based on carbon metabolism and energy conversion. Biotechnol Biofuels.

[28] Doan TTY, Obbard JP (2012). Enhanced intracellular lipid in Nannochloropsis sp via random mutagenesis and flow cytometric cell sorting. Algal Res..

[29] Lee I, Han JI (2015). Hydrothermal-acid treatment for effectual extraction of eicosapentaenoic acid (EPA)-abundant lipids from *Nannochloropsis salina*. Bioresour Technol.

[30] Gunerken E, D’Hondt E, Eppink MH, Garcia-Gonzalez L, Elst K, Wijffels RH (2015). Cell disruption for microalgae biorefineries. Biotechnol Adv.

[31] Wu C, Xiao Y, Lin W, Li J, Zhang S, Zhu J, Rong J (2017). Aqueous enzymatic process for cell wall degradation and lipid extraction from *Nannochloropsis* sp. Bioresour Technol.

[32] Scholz MJ, Weiss TL, Jinkerson RE, Jing J, Roth R, Goodenough U, Posewitz M, Gerken HG (2014). Ultrastructure and composition of the *Nannochloropsis gaditana* cell wall. Eukaryot Cell.

[33] Tan CH, Show PL, Chang JS, Ling TC, Lan JC (2015). Novel approaches of producing bioenergies from microalgae: a recent review. Biotechnol Adv.

[34] Passos F, Uggetti E, Carrere H, Ferrer I (2014). Pretreatment of microalgae to improve biogas production: a review. Bioresour Technol.

[35] Aoki J, Inoue A, Makide K, Saiki N, Arai H (2007). Structure and function of extracellular phospholipase A1 belonging to the pancreatic lipase gene family. Biochimie.

[36] Sandström AG, Wikmark Y, Engström K, Nyhlén J, Bäckvall JE (2012). Combinatorial reshaping of the Candida antarctica lipase A substrate pocket for enantioselectivity using an extremely condensed library. Proc Natl Acad Sci USA.

[37] Valverde LM, Moreno PAG, Cerdán LE, López EN, López BC, Medina AR (2014). Concentration of docosahexaenoic and eicosapentaenoic acids by enzymatic alcoholysis with different acyl-acceptors. Biochem Eng J.

[38] Huang J, Xia J, Yang Z, Guan F, Cui D, Guan G, Jiang W, Li Y (2014). Improved production of a recombinant *Rhizomucor miehei* lipase expressed in Pichia pastoris and its application for conversion of microalgae oil to biodiesel. Biotechnol Biofuels.

[39] He Y, Wu T, Wang X, Chen B, Chen F (2018). Cost-effective biodiesel production from wet microalgal biomass by a novel two-step enzymatic process. Bioresour Technol.

[40] Korman T, Sahachartsiri B, Charbonneau D, Huang G, Beauregard M, Bowie J (2013). Dieselzymes: development of a stable and methanol tolerant lipase for biodiesel production by directed evolution. Biotechnol Biofuels.

[41] Akanbi TO, Sinclair AJ, Barrow CJ (2014). Pancreatic lipase selectively hydrolyses DPA over EPA and DHA due to location of double bonds in the fatty acid rather than regioselectivity. Food Chem.

[42] Sampath C, Belur PD, Iyyasami R (2018). Enhancement of n-3 polyunsaturated fatty acid glycerides in Sardine oil by a bioimprinted cross-linked *Candida rugosa* lipase. Enzyme Microb Technol.

[43] Kahveci D, Xu X (2011). Repeated hydrolysis process is effective for enrichment of omega 3 polyunsaturated fatty acids in salmon oil by *Candida rugosa* lipase. Food Chem.

[44] Liu JY, Song YM, Qiu W (2017). Oleaginous microalgae *Nannochloropsis* as a new model for biofuel production: review and analysis. Renew Sust Energ Rev..

[45] Cesarini S, Haller RF, Diaz P, Nielsen PM (2014). Combining phospholipases and a liquid lipase for one-step biodiesel production using crude oils. Biotechnol Biofuels.

[46] Amoah J, Ho SH, Hama S, Yoshida A, Nakanishi A, Hasunuma T, Ogino C, Kondo A (2016). Lipase cocktail for efficient conversion of oils containing phospholipids to biodiesel. Bioresour Technol.

[47] Canet A, Bonet-Ragel K, Benaiges MD, Valero F (2016). Lipase-catalysed transesterification: viewpoint of the mechanism and influence of free fatty acids. Biomass Bioenerg.

[48] Hama S, Noda H, Kondo A (2018). How lipase technology contributes to evolution of biodiesel production using multiple feedstocks. Curr Opin Biotechnol.

[49] Mohammadi M, Habibi Z, Dezuarei S, Yousefi M, Ashjari M (2015). Selective enrichment of polyunsaturated fatty acids by hydrolysis of fish oil using immobilized and stabilized *Rhizomucor miehei* lipase preparations. Food Bioprod Process.

[50] Chiu SY, Kao CY, Tsai MT, Ong SC, Chen CH, Lin CS (2009). Lipid accumulation and CO_2_ utilization of Nannochloropsis oculata in response to CO_2_ aeration. Bioresour Technol.

